# The RNA-binding protein KSRP reduces asthma-like characteristics in a murine model

**DOI:** 10.1007/s00011-025-02024-5

**Published:** 2025-03-17

**Authors:** Kim-Alicia Palzer, Vanessa Bolduan, Jelena Lakus, Ingrid Tubbe, Evelyn Montermann, Björn E. Clausen, Matthias Bros, Andrea Pautz

**Affiliations:** 1 Department of Pharmacology, University Medical Center of the Johannes Gutenberg-University, Mainz, Langenbeckstr. 1, 55131 Mainz, Germany; 2 Department of Dermatology, University Medical Center of the Johannes Gutenberg-University, Mainz, Langenbeckstr. 1, 55131 Mainz, Germany; 3 Paul Klein Center for Immune Intervention, Institute for Molecular Medicine, University Medical Center of the Johannes Gutenberg-University, Mainz, Langenbeckstr. 1, 55131 Mainz, Germany

**Keywords:** KSRP, Th2 cells, Cytokines, Allergic asthma

## Abstract

**Background and objective:**

Asthma is a chronic inflammatory disease characterized by dysregulated cytokine expression. The RNA-binding protein KSRP reduces the expression of several pro-inflammatory mediators. Therefore, we investigated whether KSRP modulates Th2-associated immune responses in vivo in an ovalbumin-induced (OVA) allergic asthma model in C57BL/6 KSRP-deficient mice (KSRP^−/−^).

**Methods:**

Asthma severity in OVA-immunized wild type or KSRP^−/−^ mice was determined by airway hyperresponsiveness (AHR), structural changes of lung tissue, and OVA-specific antibody production. Cytokine expression in bronchoalveolar lavage fluid (BALF) was measured by Cytometric Bead Array (CBA) analysis. Cellular signaling pathways involved in KSRP-mediated effects in asthma pathogenesis were analyzed in vitro in cell culture models using specific inhibitors.

**Results:**

KSRP deficiency exacerbates OVA-induced allergic asthma compared to wild type mice, as indicated by increased AHR, more severe lung damage, goblet cell hyperplasia and increased OVA-specific antibody production. CBA analyses confirmed, that KSRP deficiency enhances IL-4, IL-5 and IL-13 production in BALF. The effect of KSRP on Th2-associated cytokine expression appears to be mediated by modulation of the STAT6 and NFAT signaling pathway rather than by inhibiting the stability of cytokine-encoding mRNA species.

**Conclusion:**

Our data demonstrate that KSRP dampens Th2 immune cell activity and therefore seems to be important for the pathogenesis of Th2-mediated diseases.

**Supplementary Information:**

The online version contains supplementary material available at 10.1007/s00011-025-02024-5.

## Introduction

Cytokines are important regulators of immune cell function. On the one hand, fast and robust expression of cytokines is necessary to establish a swift immune response to an invading pathogen. On the other hand, cytokine expression has to be controlled strictly to avoid an excessive immune response that may lead to tissue destruction or autoimmune reactions [1]. Expression of cytokines is controlled by transcriptional, post-transcriptional and translational mechanisms. In this regard, modulation of mRNA stability is one important post-transcriptional mechanism. Cytokine mRNAs that are regulated by RNA-binding proteins (RBP) are often characterized by AU-rich elements (AREs) located in the 3’-untranslated region (3′-UTR). These AREs serve as binding sites for different RBP that either destabilize (AUF1, TTP, KSRP) or stabilize (HuR) the cytokine transcript [2, 3].

KSRP (K homology (KH)-type splicing regulatory protein) is a single-stranded nucleic acid-binding protein with multiple functions that convergingly regulate the biogenesis and function of mRNAs by transcriptional [4], post-transcriptional [5] and translational [6] mechanisms. One of the best characterized KSRP functions is its ability to mediate rapid decay of ARE-containing mRNAs. mRNAs of pro-inflammatory mediators that possess AREs, such as tumor necrosis factor-α, interleukin (IL)-8 or interferons (IFN) are targets of KSRP-mediated mRNA decay [7]. Therefore, KSRP has been considered as an important negative regulator of pro-inflammatory gene expression [8].

Importance of KSRP for the innate immune response has been demonstrated in C57BL/6J KSRP^−/−^ mice, which are more resistant to herpes simplex virus type 1 infection compared to wild type (WT) mice due to KSRP-mediated regulation of type I IFN mRNA expression [9]. In addition, KSRP-deficient macrophages also produce increased amounts of pro-inflammatory mediators [10]. Rather limited information exists concerning the role of KSRP in adaptive immune cells. In vitro results indicate that KSRP also reduces production of T helper cell 2 (Th) 2-associated cytokines [11]. These results suggest that KSRP may be an important negative regulator of Th2 cell activity, but no one knows whether these in vitro data are relevant to immune responses in vivo.

Allergic asthma is a chronic inflammatory disease characterized by hyper-reactivity of the airways and increased infiltration of immune cells in the respiratory tract, especially of eosinophilic granulocytes and T cells [12]. The Th2 subpopulation, which generates IL-4, IL-5, IL-9 and IL-13, contributes to asthma disease pathogenesis. These mediators induce IgE production by B cells, contribute to activation and recruitment of mast cells and eosinophil granulocytes, and trigger goblet cell hyperplasia, mucus hyper-secretion and airway hyper-responsiveness (AHR) [13]. The ovalbumin (OVA)-induced asthma model is a well-established model of acute respiratory AHR and is characterized by a Th2-biased immune response [14]. Some reports in the literature postulate that RBP can be involved in asthma pathogenesis. For example, IL-4 mediated activation of signal transducer and activator of transcription 6 (STAT6) induces tristetraprolin (TTP, ZFP36) expression [15]. TTP is a RBP that reduces the expression of pro-inflammatory mRNAs such as TNF-α by decreasing its mRNA stability [16]. It has been described that the expression of members of the TTP family (ZFP36, ZFP36 L2) was reduced in T cell samples of asthma patients compared to T cells obtained from healthy donors. This may contribute to enhanced mRNA expression of IL-8, IFN-γ, MIP-1α, MIP-1β, and TNF-α [17]. Moreover, a dysregulation of ZFP36L1/L2 mRNA levels in combination with altered subcellular localization has been described in an asthma mouse model which may contribute to disease pathogenesis [17]. The human embryonic lethal abnormal vision-like protein (ELAVL1) or HuR, is a RBP known to stabilize cytokine mRNA transcripts. The Th2-associated cytokines IL-4, IL-13 and the Th2 cell transcription factor GATA3 are known HuR targets and therefore the protein could enhance Th2 cell-mediated immune reactions [18]. It has been previously described that KSRP and HuR may have an antagonistic role in the regulation of some shared target mRNAs (e.g. c-fos, inducible nitric oxide synthase) [19, 20], but it is not known whether this also applies for Th2-associated cytokines. In addition to RBP also long noncoding RNAs (e.g. MALAT1, NEAT1, etc.) and miRNAs (e.g. miR-155, miR-17–92, let-7, etc.) have been described to be involved in regulation of T cell activity in allergic asthma [21, 22].

The aim of the study was to investigate whether allergic asthma-like characteristics (AHR, lung inflammation, mucus production, allergen specific antibodies) were aggravated in KSRP deficient mice (KSRP^−/−^) compared to wild type animals due to altered expression of Th2-associated cytokines in vivo. In addition, we aimed to elucidate which molecular mechanisms and signaling pathways in KSRP-deficient immune cells may contribute to the observed phenotype.

## Materials and methods

### Animals

The housing and breeding of B6.129/B6F1-*Ksrp*^*tm1cyc*^/J mice [9] in our laboratory were described previously [11]. All animal studies were performed with 8- to 12-week-old female mice and the animal studies were approved in accordance with the German animal protection law and the guidelines for the use of experimental animals as stipulated by the Guide of Care and Use of Laboratory Animals of the National Institutes of Health. The animal studies were approved by the ethical board (23 177-07/G 17-1-061, 23 177-07/G 23-1-037).

### Murine ovalbumin-induced allergic asthma model

KSRP-deficient and WT mice were used for an OVA-induced allergic asthma model. To induce asthma in mice, sensitization with 20 µg of the model allergen OVA (Merck) was performed on days 0 and 14. For this, OVA was administered together with 1.2 mg of the adjuvant aluminum hydroxide (Thermo Fisher) by intraperitoneal (i.p.) injection while the control animals received a PBS/Alum mix. For provocation all mice were nebulized on days 28, 29 and 30 with 3% Isoflurane and challenged by intranasally (i.n.) injected 100 µg OVA (Merck). Two days later (day 32) the mice were subjected to an invasive lung function measurement. Mouse lungs were lavaged with 600 µl of sterile PBS (Gibco) and the cells of bronchoalveolar lavage (BAL) as well as fluid (BALF) were harvested for cytospins and CBA analysis, respectively. Mouse sera were collected and used for OVA-specific ELISA. Mouse lungs were used for immune-histological, flow cytometric or qRT-PCR analysis.

### Invasive lung function measurement (airway hyperresponsiveness)

AHR was measured by an invasive lung function test via the BUXCO system. On anaesthetized animals (100 mg/ml ketamine (Hameln pharma), 4 mg/ml acepromacine maleate (Sigma), 10 mg/kg xylazine (Bayer)) a tracheotomy was performed. Baseline airway reactivity of the animals was detected by nebulizing them with PBS (Gibco). Subsequently, the animals were subjected to ascending concentrations of aerolized metacholine (Sigma). The pressure difference of the airway resistance to the baseline was recorded. Data were analyzed using the BUXCO FinePoint software.

### Cytospins

After centrifugation of the BAL (10 min, 300×*g*, 4 °C) cell pellets were resuspended in 500 µl PBS and cytospins were generated. Cells were transferred onto slides. Diff-Quick staining (Medion Grifols Diagnostics) was performed according to the manufacturers´ protocol to differentiate the immune cell types.

### Flow cytometry

For flow cytometric analysis isolated lung cells (obtained by using the mouse Dissociation Kit (Miltenyi Biotec,) according to the manufacturers´ protocol) were seeded into 96-well culture plates. All cells were incubated for 30 min at 4 °C in the dark with different antibodies (supplemental Table 1). Samples were fixed with 0.7% paraformaldehyde (Roth) and assayed using an Attune NxT flow cytometer (Thermo Fisher). Data were analyzed with the Attune NxT software.

### OVA-specific ELISA

OVA-specific IgE was quantified using the mouse OVA-IgE ELISA Kit (mdbioproducts) whereas OVA-specific IgG1 ELISA was performed by using the goat anti-mouse IgG1 Biotin antibody (Southern Biotech). An IgE (mdbioproducts) or IgG1 (Sigma) serial dilution was performed as a standard and measured in a Spectra Max iD3 detector (Molecular Devices).

### Immunohistological analysis

Lungs were fixed with 4% formaline (Roth) and embedded in paraffin. Preparation of lung tissue Sections (5 µm), immunohistological hematoxylin/eosin (HE) staining as well as periodic acid-Schiff (PAS) staining was performed by the histologic core facility of the FZI. Lung pathology, cell infiltration and mucus production were assessed using the scoring parameters listed in supplementary Tables 2 and 3.

### Isolation of murine spleen cells

Primary murine spleen cells from KSRP^+/+^, KSRP^+/−^ (WT) and KSRP^−/−^ (KSRP-deficient, KO) were isolated and squeezed through a cell strainer (40 µm). After centrifugation (10 min, 300xg, 4°C), erythrocytes were lysed for 1 min by applying 1 ml lysis buffer. The reaction was stopped by adding 20 ml of cell culture medium (media see supplemental Tables 4–6).

### Isolation of murine CD4^+^ T cells

Primary murine splenic CD4^+^ T cells were isolated using the CD4^+^ T cell Isolation Kit mouse (Miltenyi Biotec, Bergisch Gladbach, Germany) as described by the manufacturer. Isolated CD4^+^ T cells were cultured in the same medium as total spleen cells.

### In vitro stimulation and inhibition

Primary murine splenic cells were stimulated with 1 µg/ml phorbol-12-myristat-13-acetat (PMA; Merck, Darmstadt, Germany) and 4 µmol/ml ionomycin (Merck) (P/I). Primary CD4^+^ T cells were polyclonally stimulated with 1 µg/ml anti-CD3 (aCD3) antibody (eBioscience) and 2 µg/ml anti-CD28 (aCD28) antibody (eBioscience). STAT6 inhibitor AS1517499 (300 nM MedChemExpress) or 100 ng/ml cyclosporin A (NFAT inhibitor, Merck) were added 1h before cells were stimulated. 24h later mRNA expression and protein expression was examined.

### mRNA stability analyses

5 × 10^5^ CD4^+^ T cells/ml were seeded into 24-well plates and stimulated polyclonally for 24 h. Then 25 µg/ml 5,6-Dichlorobenzimidazole 1-ß-D-Ribofuranoside (DRB; Sigma) were added to inhibit RNA polymerase II activity, which results in inhibition of mRNA transcription. RNAs were prepared 0, 0.5, 1, 3 and 6 h thereafter. The amount of IL-4 mRNA was determined by qRT-PCR.

### RNA isolation: reverse transcription—qRT-PCR

RNA was prepared by guanidinium isothiocyanate (GIT) chloroform extraction, as previously described in [23]. cDNA was synthesized using the High-Capacity cDNA Reverse Transcription Kit (Applied Biosystems) and gene expression was quantified in a two-step real-time RT-PCR using SYBR Green (Amplifyme SG universal mix, 7Bioscience) according to the manufacturer. The sequences of the primers are listed in supplemental Table 7. Relative mRNA expression rates were calculated using the 2^ΔΔ*C*(*T*)^ method [24] and murine GAPDH or actin was used for normalization.

### Western blot analyses

50–100 µg protein of splenic cells were separated by SDS-PAGE. Proteins were detected with specific antibodies (supplemental Table 8). As an endogenous control β-Tubulin was used. Antibody signals were visualized by enhanced chemiluminescence detection system (Thermo Fisher) and quantified with the Quantity One Software (Bio-Rad). Protein intensity was corrected by subtracting the background and normalization with β-Tubulin.

### Cytometric bead array (CBA)

Cytokine concentration was determined by Cytometric Bead Array (CBA). A serial cytokine dilution with the mouse cytokine flex set (BD Bioscience) was performed as a standard and measured in an Attune NxT flow cytometer (Thermo Fisher). Data were analyzed using the FCAP Array Analysis software (BD Bioscience).

### Statistics

Data represent means ± SEM. Statistical differences were determined using different statistical tests (t-test analyses and two-way ANOVA analysis with Bonferroni´s multiple comparison test) shown in all figure captions. Significant differences were marked and all statistical analyses were performed using GraphPad Prism 10.0 (GraphPad Software, La Jolla, CA).

## Results

### Allergic airway disease in KSRP^−/−^ mice

**Fig. 1:**
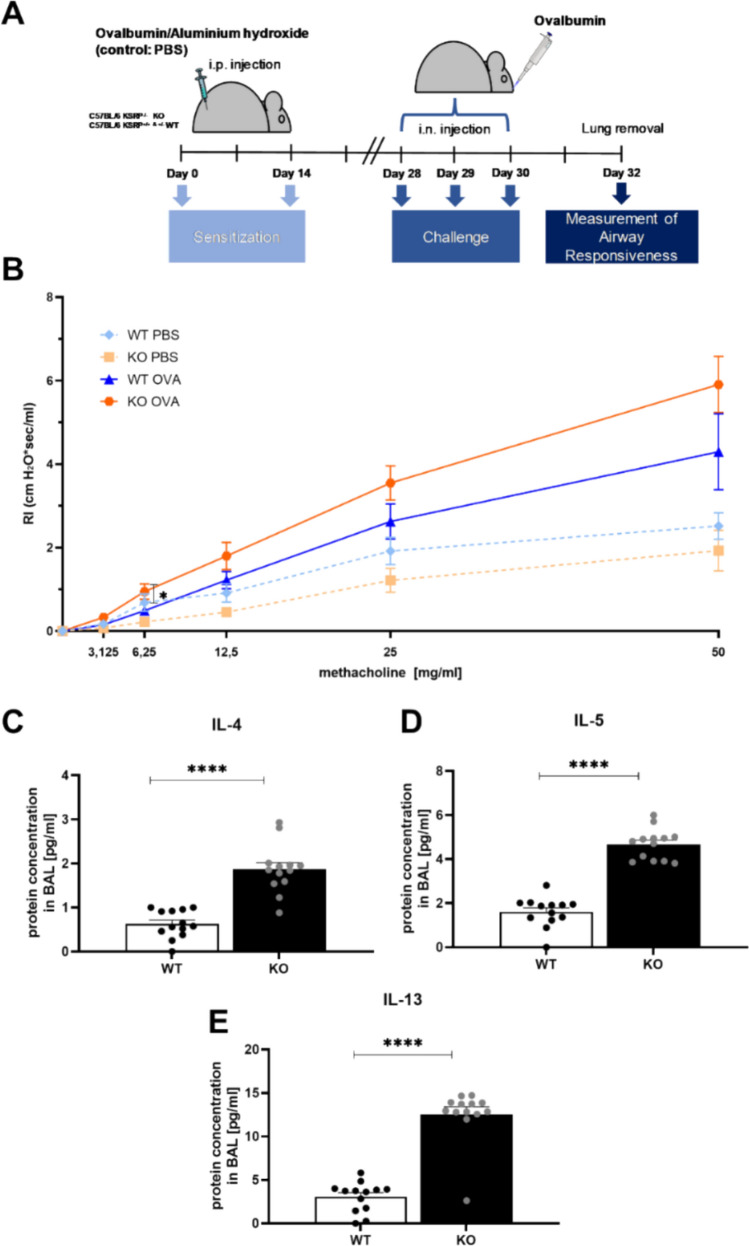
Airway hyperresponsiveness (AHR) and Th2 protein production in fluid of bronchoalveolar lavage (BALF) after sensitization/challenge of wildtype (WT: KSRP^+/+^ and KSRP^±^) and KSRP-knockout mice (KO: KSRP^−/−^) with OVA. (**A**) Scheme of OVA-induced asthma model. In this acute provocation model, animals were immunized on days 0 and 14 each with 20 µg OVA (together with 1.2 mg Alum) i.p. (control: PBS/alum). For provocation, the mice were challenged on days 28–30 each with 100 µg OVA i.n. On day 32 the mice were subjected to an invasive lung function measurement. (**B**) The baseline airway reactivity was tested for five minutes and then the airway resistance (RI) after provocation with increasing doses methacholine (3.125, 6.25, 12.5, 25 and 50 mg/ml) was recorded. The mean values ± SEM of the RI are shown compared to the baseline (n = 10–13 animals per group and genotype). After AHR measurement, lungs were flushed with PBS and the cell-free solution of the BAL was used for CBA. Shown are the mean value ± SEM of the IL-4 (**C**), IL-5 (**D**) and IL-13 (**E**) protein concentrations in the BALF of mice compared to OVA treated WT (n = 10–13 animals per genotype). Unpaired t-test was used to determine significant differences: ns, not significant, *p < 0.05, ****p < 0.0001

We used an OVA-induced asthma model to test the hypothesis that KSRP regulates the expression of Th2-associated cytokines in vivo, suggesting that a KSRP knockout might exacerbate Th2-mediated diseases. Asthma was induced in KSRP^−/−^ and WT mice as described in Fig. 1A and in the methods section.

**Fig. 2:**
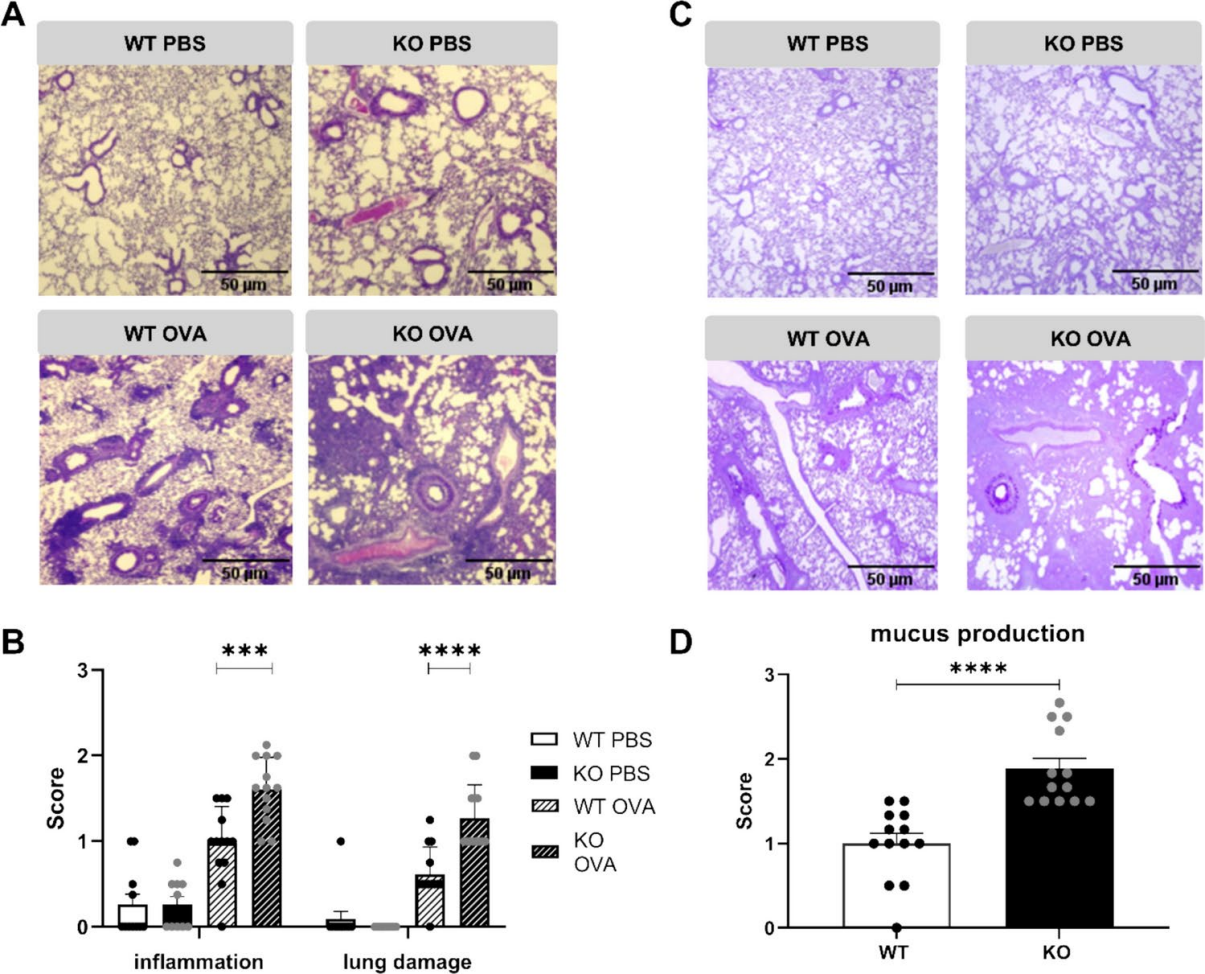
Determination of immunohistological inflammation, lung damage and mucus production of wildtype (WT: KSRP^+/+^ and KSRP^±^) and KSRP-knockout (KO: KSRP^−/−^) mice of the OVA induced asthma model. All animals were treated as described in Fig. 1. 48 h after the last challenge with OVA, sections of the lungs were stained with hematoxylin and eosin (**A**) and with Periodic-Acid-Schiff (**C**), respectively. Shown are representative tenfold enlarged lung tissue sections of control (PBS) and OVA sensitized WT and KO mice (**A**, **C**). Cell nuclei and ribosomes were stained using hematoxylin, and other cell structures like cytoplasm and collagen fibers are stained red by using eosin (**A**). Mucus-producing cells in the bronchi are stained bright violet (**C**). By using the score scheme described in supplemental Tables 7 and 8 the degrees of immune inflammation and lung damage (**B**) as well as of mucus expression were examined. Shown are the mean values ± SEM of the scores of the test groups compared to OVA treated WT scores (n = 10–13 per group per genotype). Significant differences were determined by unpaired t-test: ***p < 0.001, ****p < 0.0001

Determination of airway resistance (RI) 48 h after the last OVA challenge by invasive lung function measurement showed that ascending doses of methacholine increased airway resistance in OVA- treated animals compared to PBS control mice. The data indicate that the AHR was more severe in OVA-treated KSRP^−/−^ mice (Fig. 1B). The expression of Th2-associated cytokines in bronchoalveolar lavage fluid (BALF) was elevated two- to threefold in OVA-immunized KSRP^−/−^ mice compared to WT animals (Fig. 1C–E). H&E and PAS staining revealed an increase in immune cell infiltration, lung damage, and mucus producing goblet cells in OVA-treated animals compared to PBS-injected control mice. However, all parameters evaluated were almost twice as high in OVA-immunized KSRP^−/−^ mice compared to OVA-treated WT mice (Fig. 2). These findings support our hypothesis from the RI measurements that KSRP deficiency aggravates allergic asthma symptoms.

**Fig. 3:**
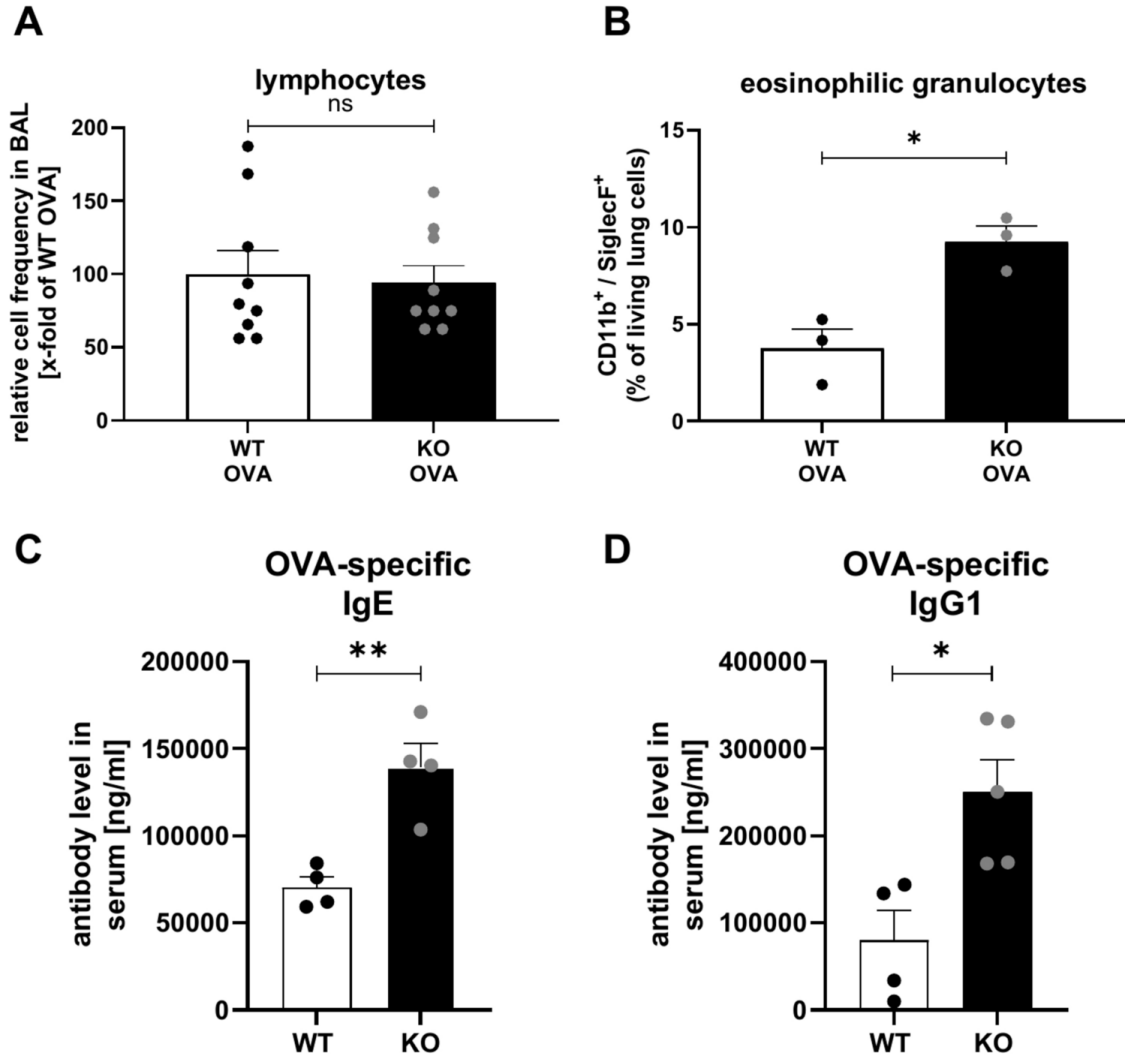
Frequency of infiltrated lymphocytes and eosinophils in BAL and lungs, respectively, of OVA-sensitized wildtype (WT: KSRP^+/+^ and KSRP^±^) and KSRP-knockout (KO: KSRP^−/−^) mice. The animals were treated as shown in Fig. 1. 48 h after the last provocation BAL, blood serum and lungs were collected. (**A**) BAL cells were centrifuged on slides and stained according to the manufacturer´s instructions by means of Diff-Quick^®^ dyeing set. The measured frequency of lymphocytes from the OVA-treated WT mice were defined as 100%. Shown are the mean values ± SEM of nine mice per genotype compared to the lymphocyte frequency of WT OVA mice (Statistics: unpaired t-test, ns not significant). (**B**) The dissociated lung cells obtained were used for flow cytometric analysis. Data denote the frequencies of eosinophilic granulocytes of living lung derived from OVA-treated animals (mean values ± SEM, n = 3 per genotype, Statistics: unpaired t-test, *p < 0.05). (**C**, **D**) Antibody concentrations (ng/ml) of OVA-specific IgE and IgG1 in blood serum was measured by ELISA. Shown are the mean values ± SEM compared to OVA-treated WT mice (n = 4–5 per genotype). Unpaired t-test was performed to identify significant differences: *p < 0.05, **p < 0.01

Increased expression of Th2-associated cytokines in OVA-immunized KSRP^−/−^ mice could be the result of enhanced lymphocyte infiltration into the lung tissue, but at least in cytospins of BAL we found no genotype-specific differences in lymphocyte numbers (Fig. 3A). IL-5 is important in asthma pathogenesis since it contributes to the recruitment and activation of eosinophils [25]. In accordance with increased IL-5 production in BALF we detected a two- to three-fold increase in eosinophil numbers in OVA-treated KSRP^−/−^ mice by flow cytometric analysis employing the eosinophil markers CD11b and SiglecF (Fig. 3B, supplemental Fig. 1). In addition, we observed enhanced mRNA expression of the eosinophil-attracting chemokine eotaxin2/CCL24 in lung tissue of OVA-treated KSRP^−/−^ mice (supplemental Fig. 1). Next, we asked whether enhanced IL-4 and IL-13 expression in KSRP^−/−^ mice modulated OVA-specific antibody production. In fact, OVA-specific IgE and IgG1 titers were significantly increased in OVA-KSRP^−/−^ mice (Fig. 3C, D). Altogether, these data demonstrate that KSRP deficiency drives Th2 immune responses in vivo leading to aggravated airway inflammation.

### Neutralization of IL-4 reduces airway inflammation in KSRP^−/−^ mice

**Fig. 4:**
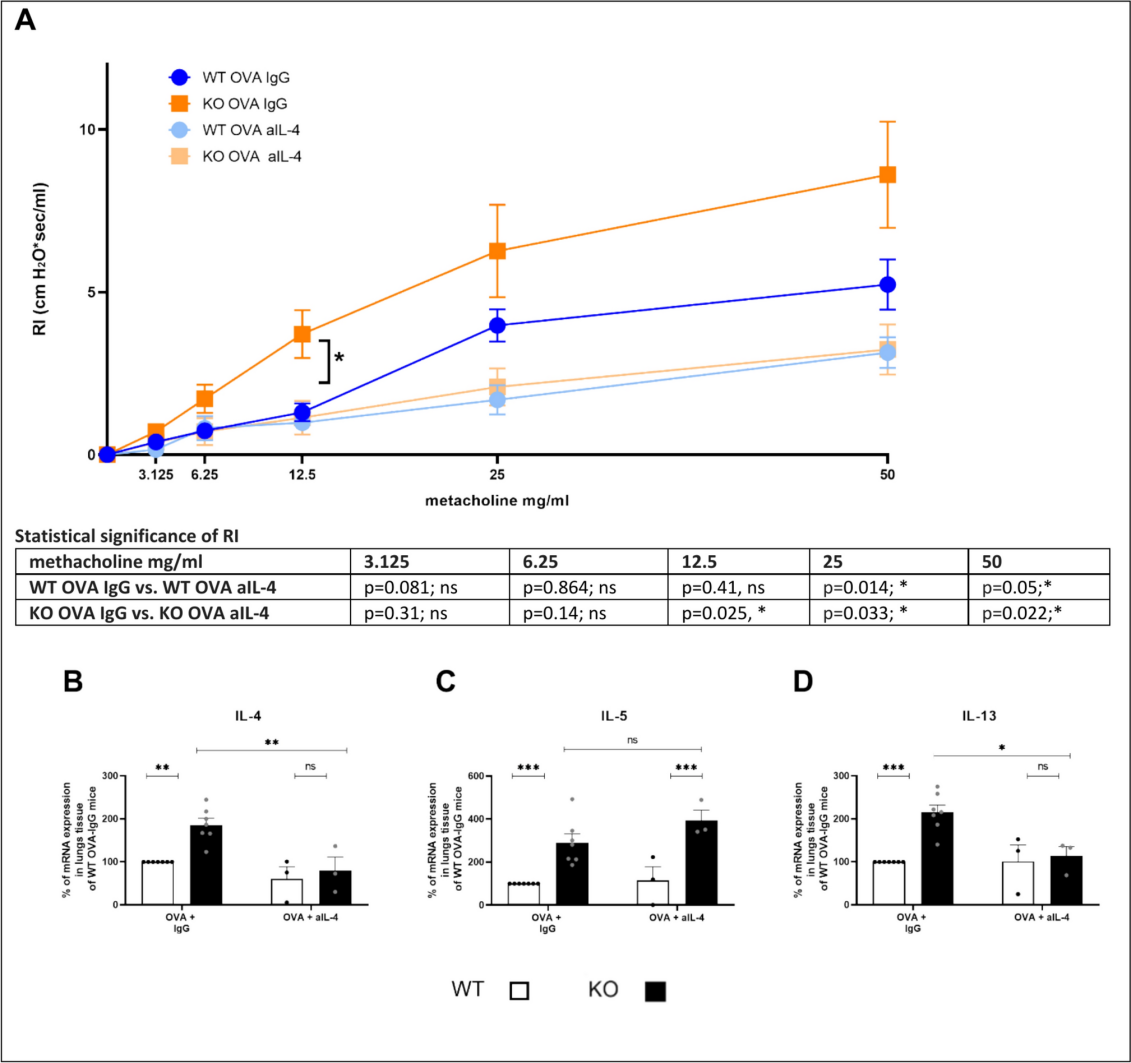

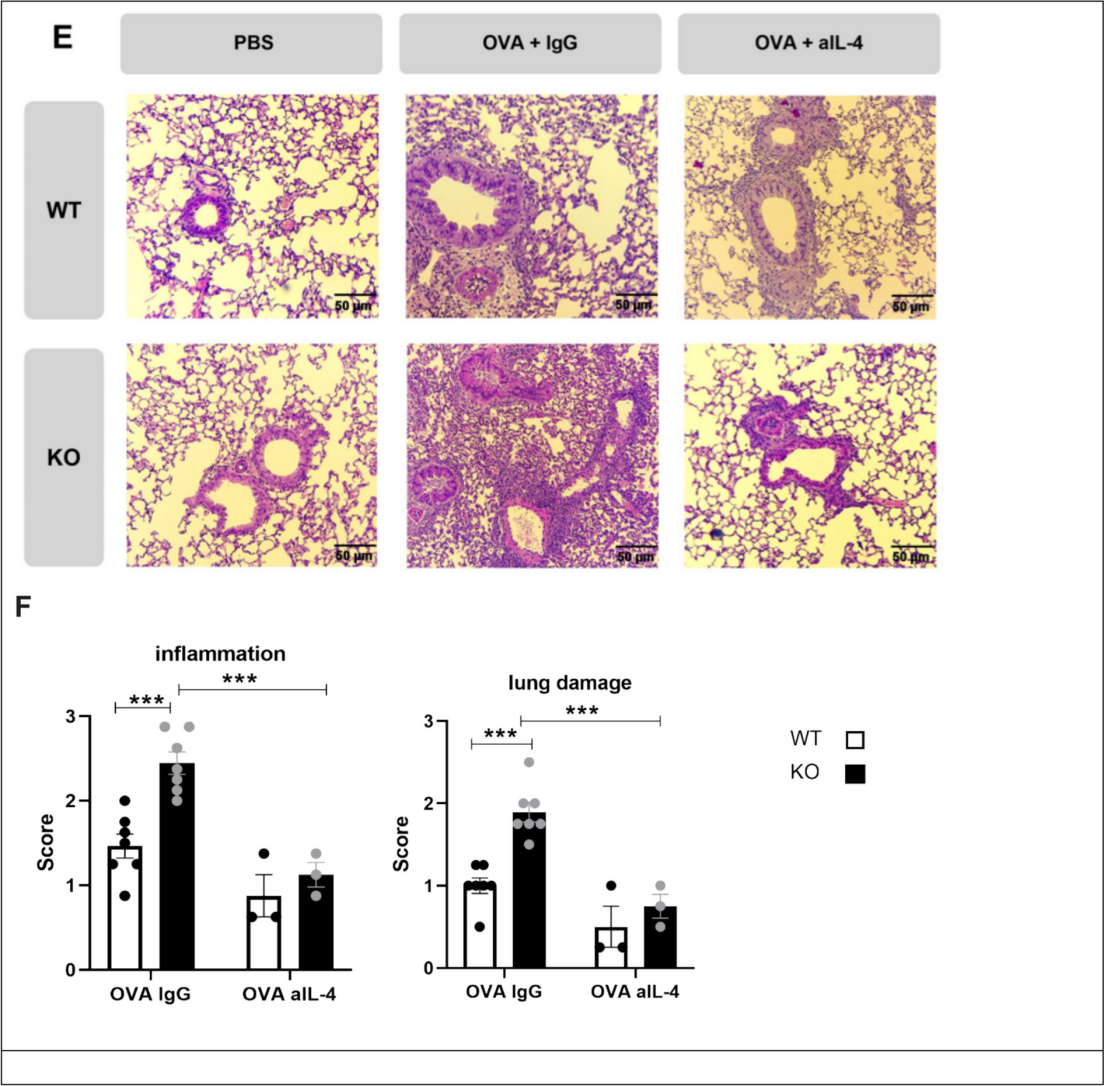
AHR, mRNA expression of Th2-associated cytokines and degree of inflammation and damage in lung tissue of OVA-challenged and with antibody treated wildtype (WT: KSRP^+/+^ and KSRP^+/−^) and KSRP-knockout (KO: KSRP^−/−^) mice. WT and KO mice were treated as described in supplemental Fig. 2. 48 h after the last challenge the airway resistance (RI) was measured (**A**). All test groups were subjected to an invasive lung function measurement. The mean values ± SEM of the RI of the test groups (n = 3–7 animals per group per genotype) are shown. The table compares RI between WT OVA IgG and WT OVA aIL-4 respectively KO OVA IgG and KO OVA aIL-4 treated mice (**B**–**D**) mRNA expression was determined by qRT-PCR by normalizing to GAPDH. Shown are the mean values of the relative IL-4 (**B**), IL-5 (**C**) and IL-13 (**D**) mRNA expressions ± SEM. mRNA expression in lung tissue of OVA-IgG control WT mice were defined as 100%. (**E**) Lung sections were stained with HE. Shown are representative tenfold enlarged lung tissue sections by using the score scheme described in supplemental Tables 7, 8 the degree of immune inflammation and lung damage (**F**) were examined. Significant differences were detected by using unpaired t-test: ns not significant, *p < 0.05, **p < 0.01, ***p < 0.001, ****p < 0.0001 (n = 3–7)

So far, our data indicate that KSRP-mediated regulation of Th2-associated cytokines can cause an aggravated airway inflammation in KSRP^−/−^ mice, but it was necessary to prove this hypothesis in vivo. Therefore, we neutralized IL-4 the main asthma-promoting T cell-derived cytokine by injecting three doses of an anti-IL-4 antibody (aIL-4), each time one hour before the intranasal OVA challenge was performed (days 28–30, supplemental Fig. 2). Airway resistance analysis demonstrated that antibody treatment reduced methacholine-induced bronchoconstriction in both WT and KSRP^−/−^ mice compared to IgG-treated control mice (Fig. 4A). The mRNA expression analysis of lung tissue showed that genotype-specific differences in OVA-induced IL-4 and IL-13 production were abolished in KSRP^−/−^ mice, while IL-5 expression was not affected by the neutralizing antibody (Fig. 4B–D). Immunohistochemistry demonstrated that KSRP-mediated effects on OVA-induced lung inflammation and damage were reduced to the levels of control mice (Fig. 4E–F).

### Determination of IL-4 mRNA stability

**Fig. 5:**
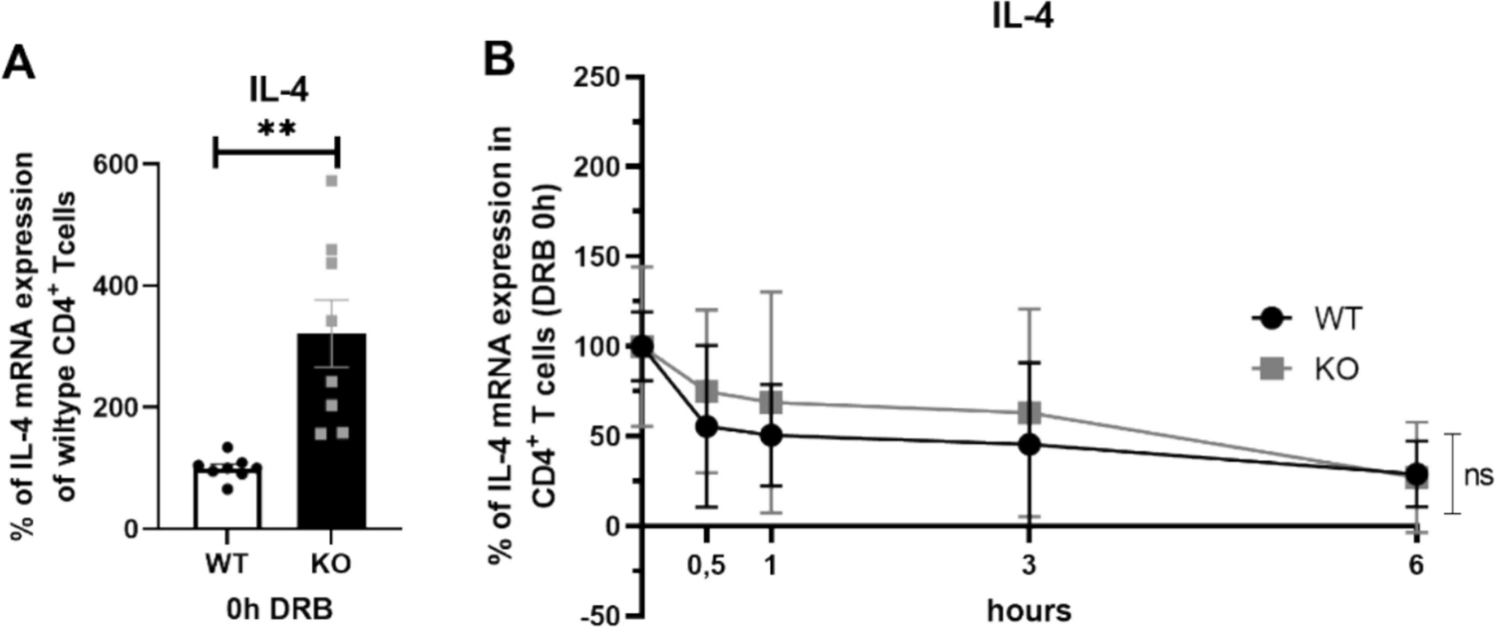
Analysis of IL-4 mRNA stability in polyclonally stimulated CD4^+^ T cells of wildtype (WT: KSRP^+/+^ and KSRP^+/−^) and KSRP-knockout (KO: KSRP^−/−^) mice. Splenic CD4^+^ T cells were polyclonally stimulated with 1 µg/ml anti-CD3 and 2 µg/ml anti-CD28 for 24h. Then, 25 µg/ml 5,6-dichloro-1-β-D-ribofuranosylbenzimidazole (DRB) was added the indicated periods of time to examine the influence of KSRP on the stability of IL-4 mRNA. RNAs were prepared and mRNA expression was determined by qRT-PCR by normalizing to actin. (**A**) Shown are the relative IL-4 mRNA expression of the KO cells compared to the WT cells at the time point of DRB addition. (**B**) Linear representation of the relative amount of IL-4 mRNA degradation where WT and KO 0h DRB controls were defined as 100%. Shown are the mean values ± SD and significant differences were detected by using unpaired t-test: ns not significant, *p < 0.05, **p < 0.01 (n = 6–8 per genotype)

Since one of the best-characterized biological functions of KSRP is its ability to regulate the stability of ARE-containing mRNAs we tested in polyclonally stimulated CD4^+^ T cells from KSRP^−/−^ or WT mice mRNA degradation of IL-4 mRNA after addition of DRB, an inhibitor of RNA polymerase II. As expected, IL-4 mRNA expression was significantly higher in KSRP^−/−^ T cells and IL-4 mRNA was rapidly degraded upon DRB addition. Nevertheless, no significant genotype-specific difference in mRNA half-life was detected (Fig. 5). Similar results were observed for IL-13 and IL-5 mRNA degradation (data not shown). These data suggest that regulation of mRNA stability is not the major mechanism responsible for KSRP-mediated regulation of Th2-associated cytokines.

### KSRP-mediated effects on STAT6

**Fig. 6:**
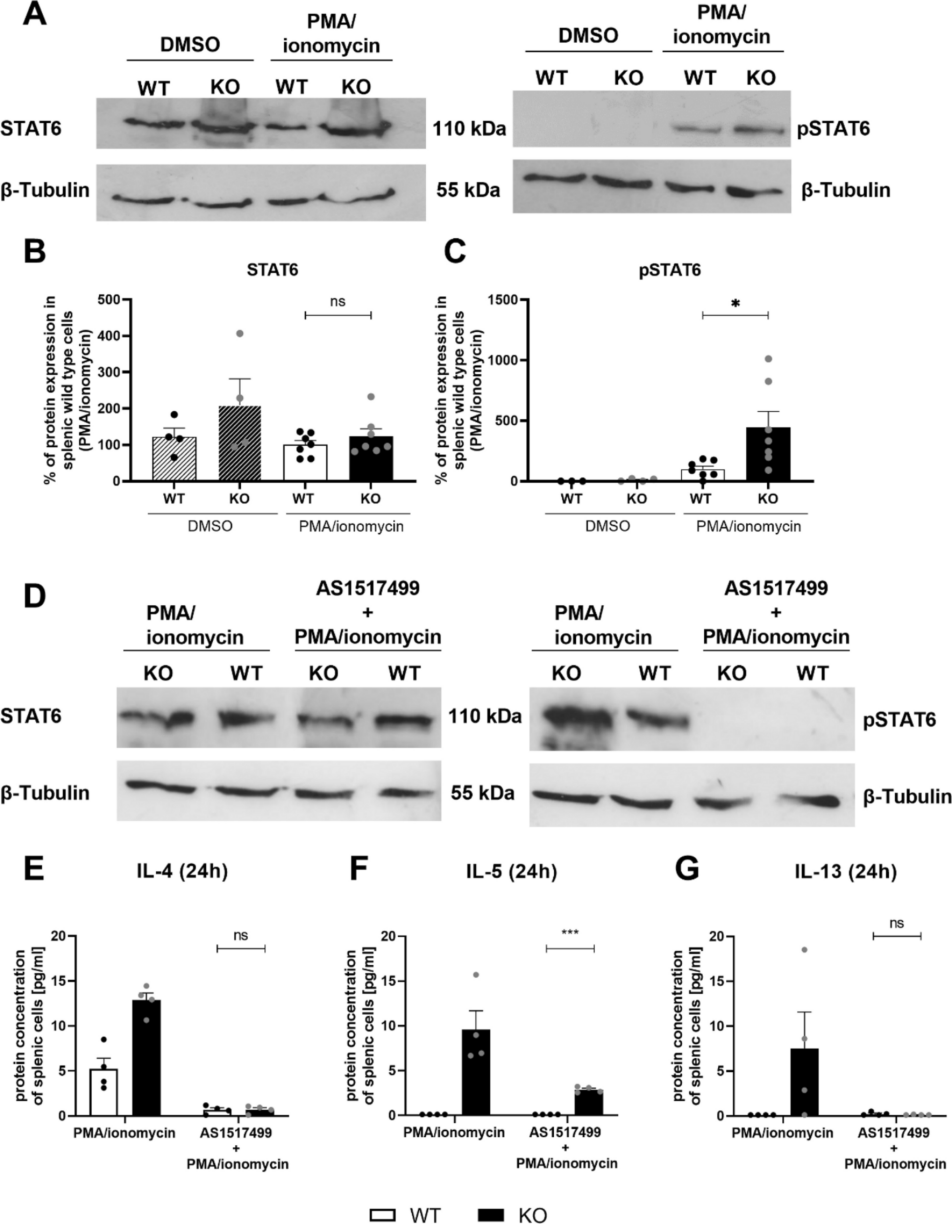
Analysis of STAT6 and phospho STAT6 (pSTAT6) protein expression and its effect on KSRP-mediated Th2 cytokine expression in murine splenic cells of wildtype (WT: KSRP^+/+^ and KSRP^+/−^) and KSRP-knockout (KO: KSRP^−/−^) mice. (**A**–**C**) Splenic cells were isolated and co-stimulated with 1 µg/ml PMA and 4 µmol ionomycin (control: DMSO). STAT6 and pSTAT6 proteins (control: β-tubulin) were detected by western blot analysis (**A**). The relative protein expression of STAT6 (**B**) and pSTAT6 (**C**) compared to protein expression of PMA/ionomycin treated WT cells was determined. Significant differences of the mean values ± SEM (n = 3–7) were determined by using unpaired t-test: ns not significant, *p < 0.05. (**D**–**G**) Splenic cells were isolated and treated with 300 nM AS1517499 for 1h. Cells were co-stimulated with PMA/ionomycin for 1 h to determine STAT6 and pSTAT6 (control: β-tubulin) protein by western blot analysis (**D**). Supernatants of cells (stimulated for 24 h) were harvested and protein concentrations (pg/ml) of IL-4 (**E**), IL-5 (**F**) and IL-13 (**G**) were determined. Shown are the mean values ± SEM compared to PMA/ionomycin treated WT cells (n = 4). Significant differences in the inhibitor group were detected by using unpaired t-test: ns not significant, ***p < 0.001

One important signaling pathway for the induction of Th2-associated cytokine expression is the IL-4/STAT 6 pathway. We therefore investigated whether the increased IL-4 production in KSRP^−/−^ cells leads to STAT6 activation and thus significantly contributes to the increase in the expression of IL-13 and IL-5. Spleen cells were stimulated with PMA/ionomycin in the presence or absence of the specific STAT6 inhibitor AS1517499. Measurements were performed 1h (Western blot) or 24h (cytometric bead array, CBA) after the start of the experiment. In comparison to WT cells, increased phosphorylation of STAT6 was detected in cells from KSRP^−/−^ mice after PMA/ionomycin stimulation (Fig. 6A, B), indicating enhanced activity of the STAT6 signaling pathway in KSRP^−/−^ cells. CBA analyses further demonstrated that inhibition of STAT6 activity by AS1517499 diminished IL-4 and IL-13 protein expression in both WT and KSRP^−/−^ cells and genotype-specific differences were also abrogated. In contrast, IL-5 protein expression was not completely reduced upon STAT6 inhibition in KSRP^−/−^ cells (Fig. 6E–G).

### KSRP-mediated effects on transcriptional regulation of Th2 cytokine expression

Some reports have demonstrated that KSRP is also able to modulate transcriptional processes [4]. Therefore, we hypothesized that KSRP may regulate transcription factors involved in Th2 cell polarization. However, flow cytometry analysis and qRT-PCR experiments showed no genotype-specific differences in the expression of the two Th2 cell-associated transcription factors GATA-3 [26] and c-MAF [27] ([28] and supplemental Figs. 3 and 4).

**Fig. 7:**
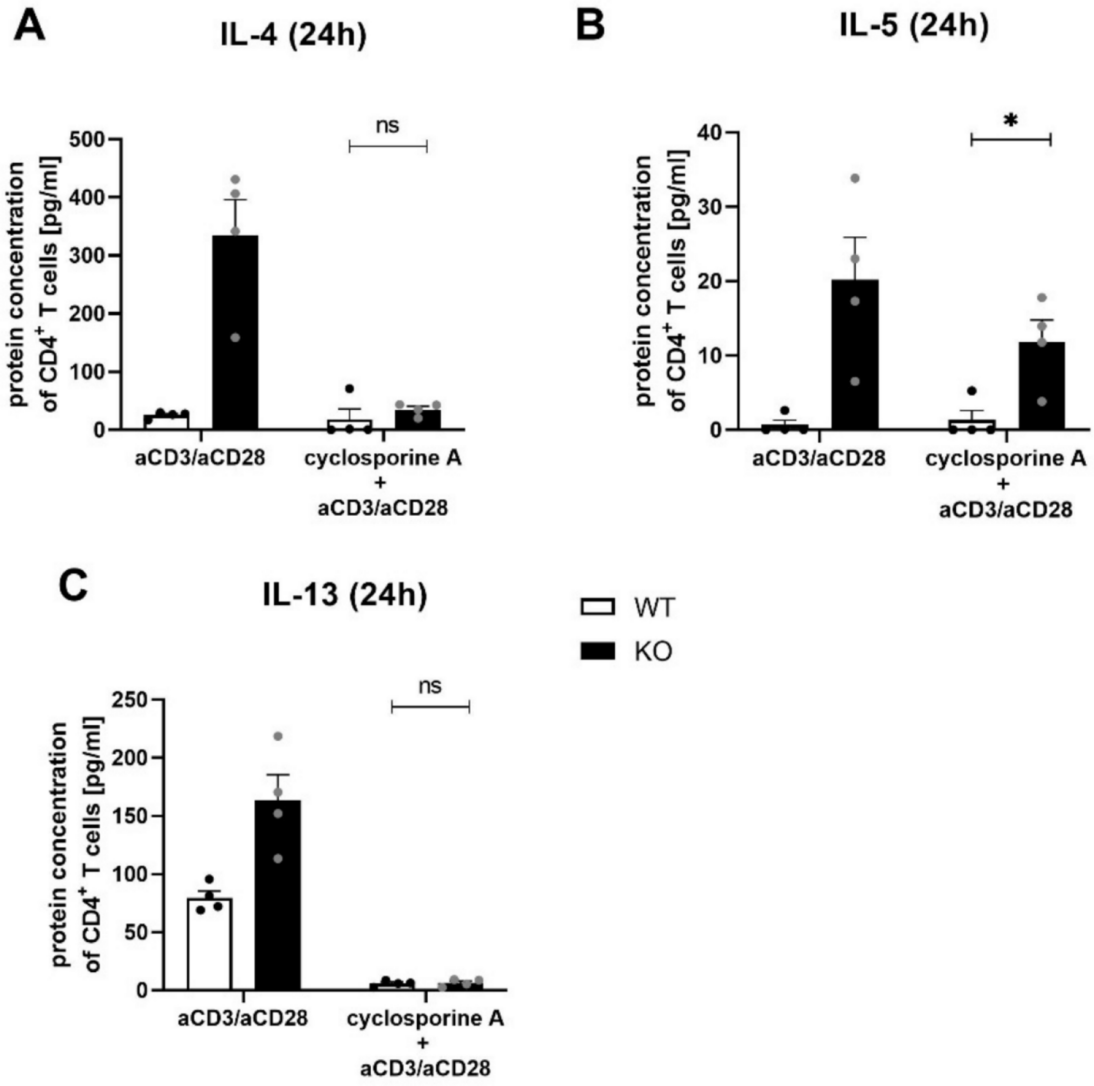
Inhibition of NFAT activity in splenic CD4^+^ T cells of wild type (WT: KSRP^+/+^ and KSRP^+/−^) and KSRP-knockout (KO: KSRP^−/−^) mice and its effects on KSRP-mediated regulation expression of Th2-associated proteins. CD4^+^ T cells isolated from spleens of WT and KO mice were treated with 0.1 µg/ml cyclosporine A to inhibit NFAT activity and were polyclonally stimulated with 1 µg/ml anti-CD3 (aCD3) and 2 µg/ml anti-CD28 (aCD28) antibody for 24 h. Supernatants were harvested and the protein concentration (pg/ml) of IL-4 (**A**), IL-5 (**B**), and IL-13 (**C**) were determined. Shown are the mean values ± SEM (n = 4 per group per genotype) compared to anti-CD3/aCD28 antibody treated WT CD4^+^ T cell. Significant differences in the inhibitor group were detected by using unpaired t-test: ns not significant, *p < 0.05

In addition, NFAT signaling is also involved in the regulation of IL-4 expression [29]. To assess its role in KSRP-dependent cytokine expression, we blocked in polyclonally stimulated CD4^+^ T cells from KSRP^−/−^ and WT mice NFAT activity with the calcineurin inhibitor cyclosporine A (CSA). CBA analysis showed that genotype-specific differences in IL-4 and IL-13 expression were almost abolished in CSA-treated WT and KSRP^−/−^ CD4^+^ T cells, while IL-5 expression persisted in KSRP-deficient cells (Fig. 7). In addition, we detected a twofold increase in NFATc1 mRNA expression in lung tissue of OVA-treated KSRP^−/−^ mice (supplemental Fig. 5). Overall, this suggests that the NFAT signaling pathway is involved in the KSRP-mediated effects on asthma progression.

## Discussion

Most mRNAs encoding dynamically regulated proteins contain ARE in their 3’UTR and their expression is often regulated not only at the transcriptional level but also post-transcriptionally. Thereby RBP, such as KSRP, TTP, AUF1, HuR, TIAR, Regnase, Roquin and Arid5a, contribute to the regulation of immune cell activity and inflammation [30].

This is the first report that demonstrates that KSRP deficiency aggravates disease symptoms in an OVA-induced, Th2 cell-mediated allergic asthma model. All characteristics of this model, such as AHR, Th2 cytokine expression, IgE antibody levels, eosinophil infiltration, and mucus hyper-production are increased in KSRP^−/−^ compared to WT mice (Figs. 1, 2, and 3). Most likely, these effects are a consequence of Th2 cell-associated cytokine production in KSRP^−/−^ mice. As is known from the literature, IL-5 might be responsible for the attraction and activation of eosinophils to the bronchi [25]. In addition to these bronchial effects, IL-5 also enhances differentiation and maturation of hematopoietic progenitor cells into eosinophils in the bone marrow (eosinophilopoiesis). This IL-5 mediated increase in eosinophil numbers might contribute to the augmented asthma-like phenotype in KSRP^−/−^ mice [31]. At the same time the increase in IL-4 and IL-13 might activate B cells [13], and IL-13 additionally may cause structural changes in lung tissue [32]. Administration of three doses of a neutralizing anti-IL4 antibody during the challenge phase reduces the aggravated allergic asthma symptoms of KSRP^−/−^ mice to the level of OVA-challenged WT animals. This indicates that KSRP-mediated regulation of these cytokines is the main reason for the stronger airway inflammation in KSRP-deficient animals, strongly suggesting that KSRP may play an important role in dampening/inhibiting Th2 cell-mediated immune responses. In addition, it would be very interesting to elucidate whether OVA-Alum sensitization alone also results in an enhanced immune response and elevation of asthma disease markers in KSRP^−/−^ mice. Such data would support our hypothesis that KSRP deficiency favors Th2 immune responses.

Since the discovery of innate lymphoid cells (ILCs), it has become clearer that more cell types and signaling pathways are involved in the pathogenesis of allergic asthma. ILCs can be divided into different subsets and ILC2 seem to contribute to allergic asthma [33]. Once activated, for example by IL-33 or IL-22, ILC2 secrete IL-5 and IL-13, contributing to AHR and airway inflammation in OVA-induced allergic asthma [34]. Currently, we cannot completely exclude that ILC2 activation contributes to the effects observed in our OVA-induced allergic asthma model in KSRP^−/−^ mice, but the fact that we do not detect increased expression of IL-33 in lung tissue samples from KSRP^−/−^ mice exposed to OVA (supplemental Fig. 6) might argue against this.

In T cells RBP-mediated regulation of mRNA stability has already been described for IL-4 and IL-13 [35–37]. Reports about post-transcriptional regulation of IL-5 mRNA stability a rare. So far, only in murine ILC2 a direct post-transcriptional regulation of IL-5 mRNA, by the RNA-BP TTP, has been demonstrated recently [38]. Since we do not detect genotype-specific differences in mRNA degradation of IL-4 or IL-13 in polyclonally stimulated CD4^+^ T cells and no degradation of IL-5 mRNA, modulation of mRNA stability by KSRP does not appear to be the main reason for the effects observed in our asthma model. Our former hypothesis, based on preliminary data, that KSRP might regulate IL-4 mRNA stability in CD4^+^ T cells [11] is not confirmed by the new data. KSRP is a protein with multiple functions. In addition to RNA, KSRP also binds to single-stranded DNA and is therefore considered as a single-stranded nucleic acid binding protein. Therefore, KSRP can also act as a transcription and splicing factor. In addition, KSRP promotes the maturation of a subset of micro (mi)RNA species, which in turn influence the expression of several genes. Therefore, there are multiple levels of KSRP-mediated regulation of gene expression [28]. Given that KSRP enhances c-myc transcription [39] and possibly regulates the transcription of multiple hematopoietic genes [4], KSRP-mediated transcriptional regulation of IL-4 expression might also be possible.

STAT6 is an important signaling molecule for Th2 cell differentiation and activation that is induced by IL-4 and IL-13 [40, 41]. The data presented in Fig. 6A, C indicate enhanced STAT6 activation in KSRP^−/−^ cells. This strongly suggests that the IL-4/JAK-STAT6 pathway is involved in KSRP-mediated effects on Th2-associated cytokine expression. Inhibition of STAT6 activation negatively regulates IL-4 and IL-13 expression (Fig. 6D–G). We used freshly isolated cells for the experiment and in these cells inhibition of STAT6 might indirectly influence, via GATA3, the expression of IL-4 and IL-13 in both wild-type and KSRP^−/−^ cells. Data from Horiuchi et. al support this hypothesis [42]. In these experiments no genotype-specific differences in IL-4 and IL-13 expression were detected. This may indicate that the KSRP-mediated changes in IL-13 expression might be rather a consequence of KSRP-dependent modulation of the IL-4/STAT6 signaling pathway and not due to a direct effect of KSRP on IL-13. The situation is different for IL-5. Since for this cytokine a genotype-specific difference persists after inhibition of the IL-4/STAT6 signaling pathway, this indicates that KSRP may directly regulate IL-5 expression independent of the IL-4/STAT6 signaling pathway.

NFAT is a family of five proteins (NFAT1-5) that are activated by calcineurin [43]. NFAT1, 2 and 4 are predominantly expressed in immune cells, and NFAT1 and 2 seem to be involved in the transcriptional regulation of IL-4 expression [43–46]. The data presented in Fig. 7 and supplemental Fig. 5 may indicate that the NFAT signaling pathway is negatively regulated by KSRP, thus reducing IL-4 and IL-13 expression, which are often co-regulated as the genes are organized in the same chromosomal region [47]. However, a direct effect of KSRP-mediated NFAT inhibition on IL-13 expression cannot be excluded since it has been demonstrated that IL-13 expression might be at least partially dependent on calcineurin activity [48–50]. Since NF-κB also binds to the IL-4 promoter region and, in cooperation with NFAT, positively regulates IL-4 expression in T cells [51], future studies will investigate the importance of this signaling pathways for KSRP-mediated effects in Th2 immune responses.

We cannot completely exclude the possibility that IL-2- also contributes to the Th2-polarising effects in KSRP^−/−^ mice, as it is known that the STAT5 pathway activated by IL-2 promotes Th2 cell activity by enhancing the accessibility of the IL-4 genomic locus or cMAF expression [52]. However, the fact that neither an increased IL-2 nor cMAF expression was found in the OVA-treated KSRP^−/−^ mice or in polyclonally stimulated CD4^+^ KSRP^−/−^ T cells compared to controls ([11] supplemental Fig. 3) argues against this hypothesis. The partial decrease in IL-5 expression (Fig. 7) could be a secondary effect of reduced IL-4 expression or a direct effect of NFAT blockade. It cannot be ruled out that KSRP regulates IL-5 expression either directly or indirectly through signaling pathways other than STAT6 or NFAT. This appear to be consistent with reports that NFAT is dispensable for the transcriptional regulation of IL-5 gene expression [53]. In addition to NFAT, the IL-5 promoter also contains transcription factor binding sites for AP-1, Oct and Elf-1 [54]. Since nothing is known in the literature about whether KSRP regulates the activity or expression of either of these factors, further research is needed to answer this question.

In particular, AP-1 represents an interesting candidate [55] as it can transcriptionally regulate a wide range of T cell-specific target genes, particularly through interaction with NFAT proteins [43, 56–59]. It is known that mRNA stability of fos, an AP-1 family member, can be regulated by KSRP and HuR [20]. Therefore, it is possible that KSRP modulates AP-1 activity in CD4^+^ T cells. In addition to IL-5 expression, this could also be important for the regulation of IL-4 expression, since cooperative binding of NFAT and AP-1 also regulates IL-4 promoter activity [60].

In conclusion, the presented data indicate that KSRP is important for the regulation of adaptive immune responses in vivo. KSRP deficiency therefore exacerbates symptoms of Th2-mediated allergic airway disease. These effects are most likely due to KSRP-mediated transcription processes and not to the regulation of mRNA stability, as one might assume based on the data in the literature. Most likely via NFAT-mediated mechanisms, KSRP primarily reduces IL-4 expression, which in turn affects STAT 6 activity and IL-13 production. Distinct Th2-associated signaling pathways seems to be affected by KSRP since KSRP-mediated regulation of IL-5 expression clearly differs from that of IL-4 and IL-13. Overall, our data suggest that KSRP is able to attenuate Th2-mediated immune responses and emphasize that KSRP is a multifunctional protein that regulates cytokine expression at different molecular levels. KSRP may be an interesting new therapeutic target in asthma therapy.

## Supplementary Information

Below is the link to the electronic supplementary material.

Supplementary file 1 (DOCX 781 KB)

## Data Availability

No datasets were generated or analysed during the current study.

## Abbreviations

RBP: RNA-binding protein
ARE: Au-rich elements
3′-UTR: 3′-Untranslated region
KSRP: KH-type splicing regulatory protein
IL: Interleukin
IFN: Interferon
WT: Wild type (KSRP^+/+^ and KSRP^±^)
KO: KSRP-deficient mice (KSRP^−^^/^^−^)
Th2: T helper cell 2
OVA: Ovalbumin
AHR: Airway hyper-responsiveness
P/I: Phorbol-12-myristat-13-acetat (PMA) and ionomycin
DRB: 5,6-Dichlorobenzimidazole 1-ß-D-Ribofuranoside
CBA: Cytometric bead array
i.p.: Intraperitoneal
i.n.: Intranasal
BAL: Bronchoalveolar lavage
BALF: Bronchoalveolar lavage fluid
HE: Hematoxylin and eosin
PAS: Periodic acid-schiff
Alum: Aluminum hydroxide
RI: Airway resistance
STAT: Signal transducers and activators of transcription
pSTAT6: Phospho-STAT6
NFAT: Nuclear factor of activated T-cells
CSA: Cyclosporine A
ILCs: Innate lymphoid cells
AP-1: Activator protein 1
