# Supplementary material for: The RNA-binding protein KSRP reduces asthma-like characteristics in a murine model
Source: Inflamm Res. 2025 Mar 17;74(1):54. doi: 10.1007/s00011-025-02024-5 (PMC11914311; doi:10.1007/s00011-025-02024-5)
Supplement: Supplementary file 1 — (DOCX 781 KB) [file 11_2025_2024_MOESM1_ESM.docx]

**Supplemental**

**The RNA-binding protein KSRP reduces the development of allergic asthma**

**Kim-Alicia Palzer^1^, Vanessa Bolduan^2^, Jelena Lakus,^3^, Ingrid Tubbe^2^, Evelyn Montermann_2_, Björn E. Clausen^3^, Matthias Bros^2^, Andrea Pautz^1^**

**Supplemental material**

Supplemental table 1: Antibodies used for FACS. To discriminate viable/dead cells were incubated with fixable viability dye (FVD eFl780, ThermoFisher) to discriminate viable/dead cell.

| **antibody** | **fluorochrom** | **clone** | **manufacturer** |
| --- | --- | --- | --- |
| Murine CD11b | SB600 | M1/70 | Thermo/LifeTechnologies |
| Murine F4/80 | eFl506 | BM8 | Thermo/LifeTechnologies |
| Murine SiglecF | Horizon BV421 | E50-2440 | BD Biociences |
| Murine Ly6G | PE | 1A8 | BD Bioscience |

Supplemental table 2: Scoring system HE staining

| **Immune inflammation** | **Score** | **Lung tissue damage** | **Score** |
| --- | --- | --- | --- |
| **No cell infiltration** | 0 | **No lung tissue damage** | 0 |
| **1. Areas of cell infiltration** | | **1. Alveolar network changes** | |
| a) peribronchiale or perivascolare | 1 | a) Single damaged | 1 |
| b) a + alveoli | 2 | b) Pronounced damage | 2 |
| c) b + bronchi/bronchioles | 3 | c) Severly damaged, fluid in the lung with infiltrated cells, hyaline membrane | 3 |
| **2. Percentage of peribronchial & perivascular cell infiltration** | | **2. Bronchi/bronchioles changes** | |
| a) ≤ 10% cell infiltartion | 1 | a) Bronchi sporadically altered, slightly thickened musculature, slightly disturbed cilia | 1 |
| b) 30% cell infiltration | 2 | b) a+ Bronchi are slightly constricted | 2 |
| c) > 50% cell infiltration | 3 | c) Bronchi severely altered, hypertrophy of the smooth muscles | 3 |
| **3. Percentage of cell-infiltrated alveoli** | |  |  |
| a) < 10% of alveoli | 1 |  |  |
| b) 10-50% of alveoli | 2 |  |  |
| c) > 50% of alveoli | 3 |  |  |
| **4. Percentage of cell-infiltrated bronchi/bronchioles** | |  |  |
| a) < 10% of bronchi/bronchioles | 1 |  |  |
| b) 10-50% of bronchi/bronchioles | 2 |  |  |
| c) > 50% of bronchi/bronchioles | 3 |  |  |

Supplemental table 3: Scoring system PAS staining

| **Mucin expression** | **Score** |
| --- | --- |
| **No mucin expression** | 0 |
| 1**. Area of mucin expression** | |
| a) bronchi/bronchioles | 1 |
| b) a + peribronchiale | 2 |
| c) b + alveoli | 3 |
| **2. Percentage of mucin-expressing bronchi** | |
| a) < 10% of bronchi | 1 |
| b) 10-50% of bronchi | 2 |
| c) > 50% of bronchi | 3 |
| **3. Percentage of mucin expression in representative bronchi** | |
| a) < 10% mucin in representative bronchi | 1 |
| b) 10-50% mucin in representative bronchi | 2 |
| c) > 50% mucin in representative bronchi or > 30% mucin expression peribronchiale | 3 |

Supplemental table 4: Composition of the isolation medium for primary cells.

| **reagent** | **concentration** | **company** |
| --- | --- | --- |
| Sterile PBS |  | Gibco |
| fetal calf serum (FBS) | 1% | PAN-Biotech, Aidenbach, Germany |
| ethylenediaminetetraacetic acid (EDTA) | 2 mM | AppliChem |

Supplemental table 5: Composition of the erythrocytes lysis buffer (pH 7,5).

| **reagent** | **concentration** | **company** |
| --- | --- | --- |
| KHCO | 10 mM | Merck |
| ammoniumchloride | 155 mM | Roth |
| ethylenediaminetetraacetic acid (EDTA) | 100 µM | AppliChem |

Supplemental table 6: Composition of the (Iscove's Modified Dulbecco's Medium) IMDM-medium for primary cells.

| **reagent** | **concentration** | **company** |
| --- | --- | --- |
| IMDM |  | Sigma-Aldrich |
| fetal calf serum (FBS) | 5% | PAN-Biotech, Aidenbach, Germany |
| Penicillin-streptomycin | 10 mg/ml | Sigma-Aldrich, Deisenhofen, Germany |
| L-glutamine | 200 mM | Sigma |
| ethylenediaminetetraacetic acid (EDTA) | 2 mM | AppliChem |
| β-mercaptoethanol | 50 µM | Gibco |

Supplemental table 7: Oligonucleotides used for qRT-PCR

| **Oligonucleotide** | **Sense** | **Antisense** |
| --- | --- | --- |
| mu actin | CGAGCACAGCTTCTTTGCAG | CGATGGAGGGGAATACAGCC |
| mu GAPDH | TTCACCACCATGGAGAAGGC | GGCATGGACTGTGGTCATGA |
| Luciferase | AAA AAG TTG CGC GGA GGA G | TTT TTC TTG CGT CGA GTT TTC C |
| **Immune relevant genes** | | |
| mu IL-4 | ACAGGAGAAGGGACGCCAT | GAAGCCCTACAGACGAGCTCA |
| mu IL-5 | GCTCTGTTGACAAGCAATGAGACG | CTCTTGCAGGTAATCCAGGAACTG |
| mu IL-13 | AGACCAGACTCCCCTGTGCA | TGGGTCCTGTAGATGGCATTG |
| mu IL-33 | AACTCCAAGATTTCCCCGGC | CCAGAACGGAGTCTCATGCA |
| mu CCL24 | TGTGACCATCCCCTCATCTTG | GCAAACTTGGTTCTCACTGCC |
| mu c-MAF | AGCAGTTGGTGACCATGTCG | TGGAGATCTCCTGCTTGAGG |

All oligonucleotides were purchased from Sigma.

Supplemental table 8: Antibodies used for western blot

| **antibody** | **size**  **(kDa)** | **manufacturer** | **Diluation** | **buffer** |
| --- | --- | --- | --- | --- |
| **Primary antibodies** | | | | |
| β-Tubulin | 55 | Sigma | 1:10000 | 5% milk powder |
| STAT6 | 110 | Cell Signaling Technology | 1:1000 |  |
| Phospho-STAT6 | 110 | Cell Signaling Technology | 1:1000 |  |
| **Secondary antibodies** | | | |  |
| Anti-rabbit |  | Sigma  (A0545) | 1:10000 |  |
| Anti-mouse |  | Sigma  (A9044) | 1:10000 |  |

**Supplemental data**

| **A**  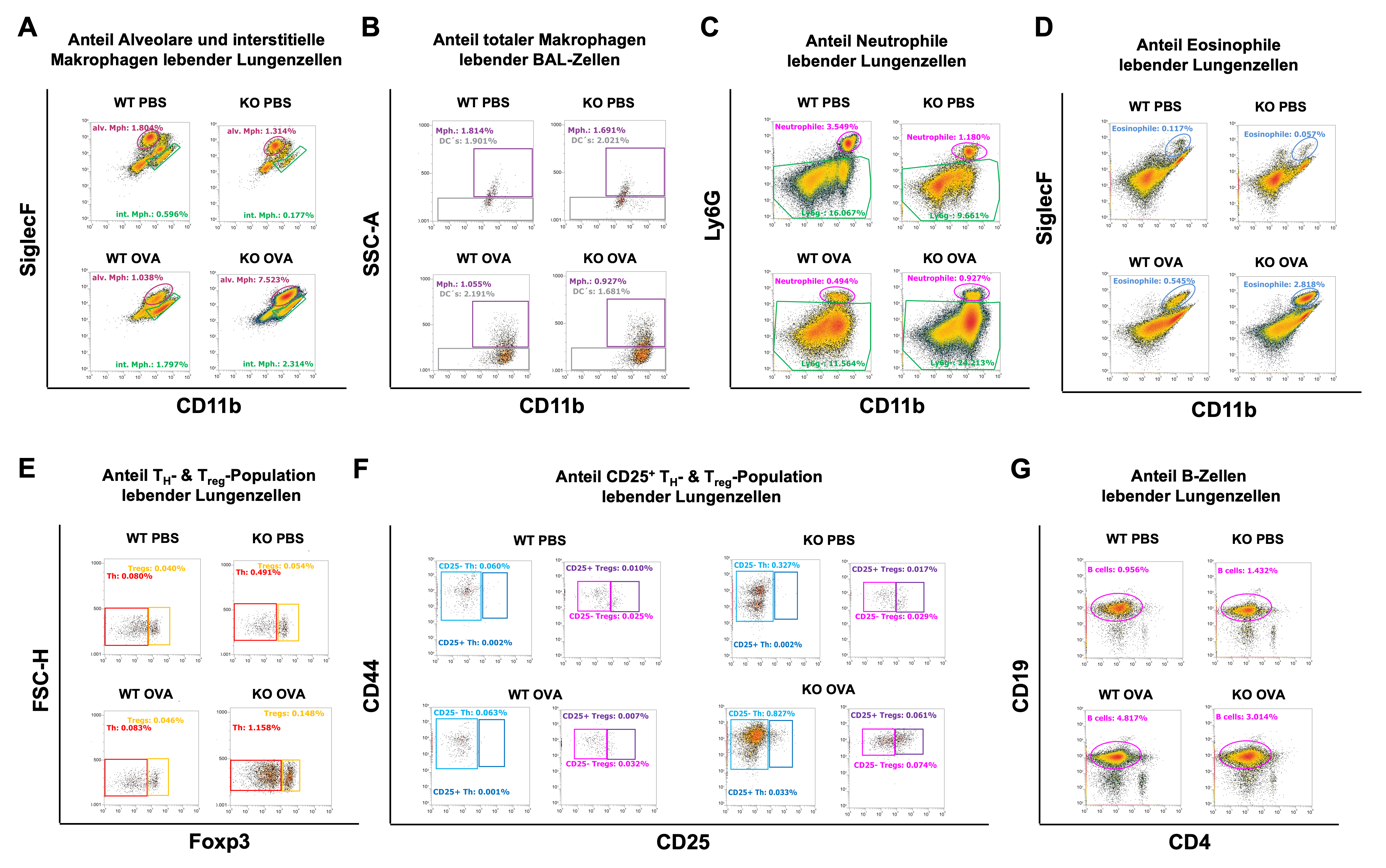 | **Suppl. Figure 1:**  **A) Gating strategy to analyze eosinophil concentrations in BALF**  **B) Eotaxin (CCL24) mRNA expression in lung tissue of with OVA sensibilized wildtype (WT: KSRP^+/+^ and KSRP^+/-^) and KSRP-knockout (KO: KSRP^-/-^) mice.** The animals were treated as shown in Fig. 1. 48 hours after the last challenge the lungs were removed. RNAs were prepared and mRNA expression was determined by qRT-PCR by normalizing to GAPDH. Shown are the mean values of the relative eotaxin mRNA expressions ± SEM. Eotaxin mRNA expression in lung tissue of with OVA treated WT mice were defined as 100%. Significant differences were detected by using unpaired t-test: ** p<0.01 (n=12-13 per genotype). |
| --- | --- |
| **B**   |  |

| 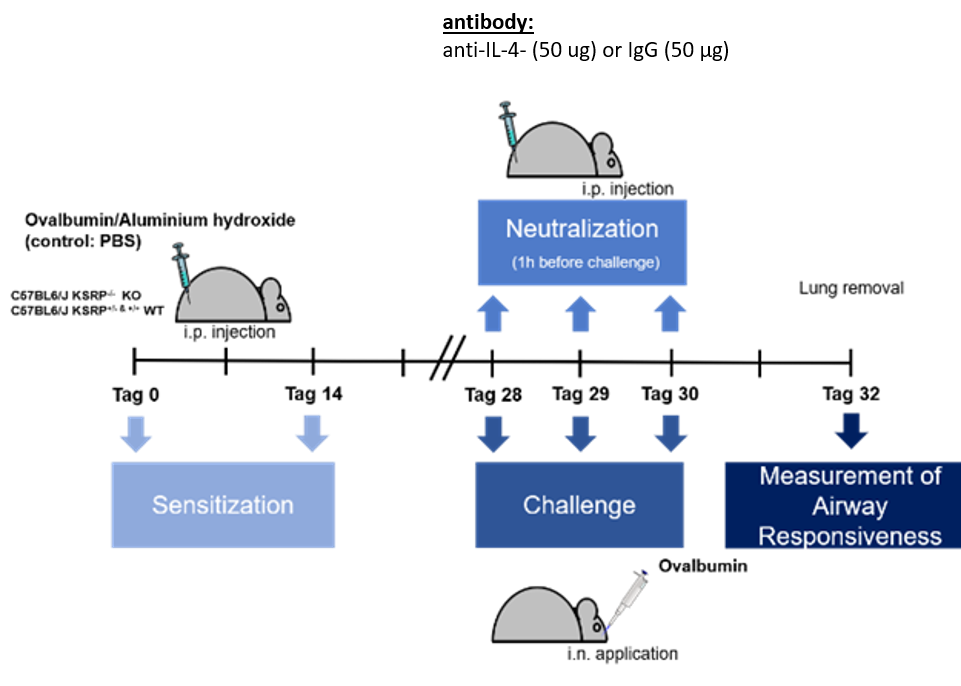 |
| --- |
| **Suppl. Figure 2: Schematic representation of the application of neutralizing antibodies in the course of a murine acute OVA-induced asthma model.** To induce asthma mice were immunized on day 0 and 14 with 20 µg OVA (together with 1.2 mg Alum) i.p. while control animals received a PBS/alum mix. For neutralizing asthma-relevant cytokines on day 28, 29 and 30 mice were treated with 50 µg anti-IL-4 antibody (control: IgG) . 1 hour after neutralization mice were challenged with 100 µg OVA i.n. On day 32 the mice were subjected to an invasive lung function measurement and the lungs were removed |

|  | **Suppl. Figure 3: cMAF mRNA expression in lung tissue of with OVA sensibilized wildtype (WT: KSRP^+/+^ and KSRP^+/-^) and KSRP-knockout (KO: KSRP^-/-^) mice.** The animals were treated as shown in Fig. 1. 48 hours after the last challenge the lungs were removed. RNAs were prepared and mRNA expression was determined by qRT-PCR by normalizing to GAPDH. Shown are the mean values of the relative cMAF mRNA expressions ± SEM. cMAF mRNA expression in lung tissue of with OVA treated WT mice were defined as 100%. Significant differences were detected by using unpaired t-test: ns not significant (n=8-9 per genotype). |
| --- | --- |

|  | **Suppl. Figure 4: GATA3 mRNA expression in lung tissue of with OVA sensibilized wildtype (WT: KSRP^+/+^ and KSRP^+/-^) and KSRP-knockout (KO: KSRP^-/-^) mice.** The animals were treated as shown in Fig. 1 48 hours after the last challenge the lungs were removed. RNAs were prepared and mRNA expression was determined by qRT-PCR by normalizing to GAPDH. Shown are the mean values of the relative GATA3 mRNA expressions ± SEM. GATA3 mRNA expression in lung tissue of with OVA treated WT mice were defined as 100%. Significant differences were detected by using unpaired t-test: ** p<0.01 (n=12-13 per genotype). |
| --- | --- |

|  | **Suppl. Figure 5: Analysis of NFATc1 mRNA expression in lung tissue of with OVA sensibilized wildtype (WT: KSRP^+/+^ and KSRP^+/-^) and KSRP-knockout (KO: KSRP^-/-^) mice.** The animals were treated as shown in Fig. 1. 48 hours after the last challenge the lungs were removed. RNAs were prepared and mRNA expression was determined by qRT-PCR by normalizing to GAPDH. Shown are the mean values of the relative NFATc1 mRNA expressions ± SEM. NFATc1 mRNA expression in lung tissue of with OVA treated WT mice were defined as 100%. Significant differences were detected by using unpaired t-test: *** p<0.001 (n=12 per genotype). |
| --- | --- |

|  | **Suppl. Figure 6: IL-33 mRNA expression in lung tissue of with OVA sensibilized wildtype (WT: KSRP^+/+^ and KSRP^+/-^) and KSRP-knockout (KO: KSRP^-/-^) mice.** The animals were treated as shown in Fig. 1. 48 hours after the last challenge the lungs were removed. RNAs were prepared and mRNA expression was determined by qRT-PCR by normalizing to GAPDH. Shown are the mean values of the relative IL-33 mRNA expressions ± SEM. IL-33 mRNA expression in lung tissue of with OVA treated WT mice were defined as 100%. Significant differences were detected by using unpaired t-test: ** p<0.01 (n=12-13 per genotype). |
| --- | --- |
